# Prevalence and predictors of undernutrition among infants aged six and twelve months in Butajira, Ethiopia: The P-MaMiE Birth Cohort

**DOI:** 10.1186/1471-2458-10-27

**Published:** 2010-01-20

**Authors:** Girmay Medhin, Charlotte Hanlon, Michael Dewey, Atalay Alem, Fikru Tesfaye, Bogale Worku, Mark Tomlinson, Martin Prince

**Affiliations:** 1 Aklilu Lemma Institute of Pathobiology, Addis Ababa University, Addis Ababa, Ethiopia; 2 Department of Psychiatry, Faculty of Medicine, Addis Ababa University, Addis Ababa, Ethiopia; 3 King's College London (Institute of Psychiatry), Health Service and Population Research Department, London, UK; 4 Department of Reproductive Health and Nutrition, School of Public Health, Addis Ababa University, Addis Ababa, Ethiopia; 5 Department of Paediatrics and Child Health, Faculty of Medicine, Addis Ababa University, Addis Ababa, Ethiopia; 6 Department of Psychology, Stellenbosch University, Matieland, South Africa

## Abstract

**Background:**

Child undernutrition is a major public health problem in low income countries. Prospective studies of predictors of infant growth in rural low-income country settings are relatively scarce but vital to guide intervention efforts.

**Methods:**

A population-based sample of 1065 women in the third trimester of pregnancy was recruited from the demographic surveillance site (DSS) in Butajira, south-central Ethiopia, and followed up until the infants were one year of age. After standardising infant weight and length using the 2006 WHO child growth standard, a cut-off of two standard deviations below the mean defined the prevalence of stunting (length-for-age <-2), underweight (weight-for-age <-2) and wasting (weight-for-length <-2).

**Results:**

The prevalence of infant undernutrition was high at 6 months (21.7% underweight, 26.7% stunted and 16.7% wasted) and at 12 months of age (21.2% underweight, 48.1% stunted, and 8.4% wasted). Significant and consistent predictors of infant undernutrition in both logistic and linear multiple regression models were male gender, low birth weight, poor maternal nutritional status, poor household sanitary facilities and living in a rural residence. Compared to girls, boys had twice the odds of being underweight (OR = 2.00; 95%CI: 1.39, 2.86) at 6 months, and being stunted at 6 months (OR = 2.38, 95%CI: 1.69, 3.33) and at 12 months of age (OR = 2.08, 95%CI: 1.59, 2.89). Infant undernutrition at 6 and 12 months of age was not associated with infant feeding practices in the first two months of life.

**Conclusion:**

There was a high prevalence of undernutrition in the first year of infancy in this rural Ethiopia population, with significant gender imbalance. Our prospective study highlighted the importance of prenatal maternal nutritional status and household sanitary facilities as potential targets for intervention.

## Background

There is an ongoing worldwide effort focused on the complete eradication of extreme poverty and hunger [1]. However, the burden of undernutrition is still a major public health problem especially in resource poor countries [2,3]. Ninety percent of the world's stunted children live in 36 developing countries [3,4]. Undernutrition remains a major cause of disability and mortality [5], ranked as the top cause of global burden of disease [6] and underlying 53% of deaths in children under five years [2,7] The potential negative impact of child undernutrition goes beyond the individual, affecting society and future generations [8-10].

Despite an encouraging global downward trend in the prevalence of stunting, the progress is not uniform across countries [2,11] According to global estimates taking the most recent available data for the years 2000-2006, the prevalence of underweight and stunting among under five children in sub-Saharan Africa were 28% and 38% respectively, while among least developed countries in general it was 35% and 42% respectively [12]. In Ethiopia there was an increasing trend of child undernutrition between 1983 and 1998 with significant variability between regions [13]. However, since 1998, four consecutive countrywide surveys showed a decreasing trend in the prevalence of underweight and stunting in all age and sex categories [14]. More recent estimates show this downward trend continuing [15]. Nevertheless, the prevalence of underweight (38%) and stunting (47%) among under five children remain substantial [12].

Epidemiological studies conducted in developing countries have identified several factors associated with undernutrition, including low parental education, poverty, low maternal intelligence, food insecurity, maternal depression, rural residential area and sub-optimal infant feeding practices [2,4,13,16-20]. Lack of maternal autonomy within the family is also recognised as a key determinant of child undernutrition [21] Analysis of data from three consecutive welfare monitoring surveys in Ethiopia over the period 1996-1998 [22] identified low household resources, lower parental education, high food prices and low maternal nutritional knowledge as key determinants of growth faltering. A study focused on the southern region of the country [23] identified low socioeconomic status of household, low maternal education, short previous birth interval, having many children aged under five years and older age of infant as risk factors for child undernutrition. The possible reasons for the high prevalence of undernutrition in the age group of 12-23 months was hypothesised to be a combination of insufficient and inappropriate supplementary foods, and recurrent infections such as diarrhoea due to poor sanitation [13]. In a one year follow-up of a large birth cohort in western Ethiopia, good sanitary conditions, availability of a water supply, increased family income and maternal literacy were associated with weight gain while the traditional surgical practices of uvulectomy and milk teeth extraction were both associated with reduction of infant weight [24]. Male sex has also been identified as one of the risk factors for undernutrition among Ethiopian children [14,15].

In this context, the objective of our study was to describe the prevalence of undernutrition of infants aged 6 and 12 months and associated risk factors, in a population based cohort: the Perinatal Maternal Mental Disorder in Ethiopia (P-MaMiE) study [25].

## Methods

### The setting

This study was conducted in the Butajira demographic surveillance site (DSS) which is located in the Meskan, Mareko and Silti Districts, Gurage Zone, in the Southern Nations, Nationalities and Peoples Regional state (SNNPR) of Ethiopia. The district capital, Butajira, is located 135 km south of the capital city, Addis Ababa, and 50 km to the west of Zway town in the Rift valley. In 2007 Butajira town had 33,393 inhabitants with a 1:1.03 male to female ratio [26]. Currently, Butajira has one hospital, one health centre and several privately owned drug stores and clinics. Various ethnic groups such as Meskan, Mareko, Silti and Sodo, live in the district. Islam and Ethiopian Orthodox Christianity have most followers relative to other religions. The livelihood of the residents is based on mixed farming. Khat (*Catha edulis Forsk*) and chilli-peppers are the main cash crops, while maize and "false banana" or Ensete (*Ensete ventricosun*) are the main staples. Due to high population density and intermittent drought, especially in the lowland area, a proportion of the population has been affected by food insecurity, leading to reliance on food aid provided through the World Food Programme.

### The Butajira Demographic and Surveillance Site (DSS)

The DSS in Butajira was established in 1986 with the main objective of generating continuous and valid data on vital statistics to be used for research and interventions [27]. The DSS includes nine rural administrative sub-districts from different ecological zones and one urban sub-district in Butajira town, which were selected using the probability proportional to size (PPS) method from Meskan and Mareko district http://www.butajira.org. Each DSS sub-district has one or two full-time employed enumerators. These are residents of their respective sub-districts who visit every household once every three months to collect data on vital events, allowing calculation of the exact population of the DSS.

Health posts and elementary schools are located within each sub-district and are reasonably accessible (a maximum of 5 km walk). During our data collection the majority of these health posts were staffed with one male community health agent (CHA) with three to six months training in primary health care delivery.

### Ethical considerations

Prior to the first interview women were informed about the objective of the study and provided informed consent. Arrangements were made for the study project to pay all health related expenses of the mothers and children participating in the study. The study was granted ethical approval from the Ethics Committee of the Institute of Psychiatry, King's College London, and National Ethics Review Committee based in the Ethiopian Science and Technology Agency.

### Study design and participants

This paper is part of the P-MaMiE cohort study designed to assess the predictors of short and intermediate term infant health and nutritional outcomes [25]. The sample size was determined to address objectives of the P-MaMiE project other than those addressed within this paper. Eligible women were between the ages of 15 and 49 years, able to speak Amharic (the official language of Ethiopia), living in the DSS and in the third trimester of pregnancy during the study recruitment period (July 2005 to February 2006). The women were identified by DSS enumerators in the course of their 3-monthly surveillance interviews and, after giving informed consent, were interviewed by the project data collectors. These data collectors were women who had completed high school education and who worked exclusively on the P-MaMiE project. The project data collectors administered questionnaires covering all the pregnancy and post-partum variables. The data collectors, DSS enumerators and health post CHAs were trained to accurately carry out anthropometric measurements.

#### Birth weight

In six of the nine rural sub-districts, CHAs were trained to measure birth weight using SECA 725 scales measuring to an accuracy of 10 g. The CHAs live and work in the sub-district and are well-known to the women. After giving birth, participating women were requested to inform the CHA of the birth so as to enable the neonate to be weighed within 24 to 48 hours of birth. Birth weights were not measured in the remaining four sub-districts as no suitable health worker was available.

### Delivery circumstances

A separate questionnaire was administered to women at the time of measuring birth weight or in the first few days after birth in order to obtain information on stillbirths, prolonged labour, pre-lacteal feeding, withholding of colostrum, and the timing of initiation of breast-feeding. Timing of initiation of breastfeeding was recorded using three categories (< 1 hour, 1-8 hours, > 8 hours following delivery) and for this analysis we combined the last two categories. In the sub-districts where birth weight was not measured, the questionnaire was administered by the project data collectors, having been informed by the traditional birth attendant or DSS enumerators that the woman had given birth. As women in this setting were unable to report their gestation at delivery with any accuracy we were unable to assess preterm delivery. It was not feasible to use last menstrual period (LMP) to estimate gestational age in this cohort because the mothers could not reliably date their LMP.

### Composite risk factors (or scales)

Including numerous individual items in a model enables measurement of the effect of the specific detailed item on the outcome which might be helpful in designing interventions. On the negative side, there is a higher likelihood of getting false positive associations as the result of multiple tests. To overcome the latter problem without significant compromise of the first issue, we created the following four composite scores from items defining the same concept by adding responses of identified variables giving equal weights to each item. Each composite variable was used as a continuous variable:

(1) Poor sanitary conditions: not having a toilet facility, not having safe water, disposing of rubbish on the field). We aggregated these three variables since all of them are known risk factors of undernutrition in Ethiopia although their internal consistency is relatively low (Cronbach alpha = 0.49).

(2) Maternal autonomy: decision-making power reflecting whether the woman is allowed to act without asking permission from her husband (to sell crops, to spend household money, attend meetings like women's groups, buy medication for herself or her children, attend a health institution for health education or for medical examination). The resulting scale had a Cronbach alpha value of 0.93.

(3) Availability of support to the mother: able to visit friends, enough help at home, enough help in looking after children other than the index child, enough help from husband and no violence towards her. This scale had a Cronbach alpha value of 0.47 which is relatively low. However, these items measure quite different sources of support and we would not expect them to correlate highly.

(4) Poverty index: non-literate wife, non-literate husband, do not own radio, do not own bed, do not possess valuable goods like gold and jewellery, own their home, own large animals, own small animals, animals spend the night within the home, home has a window. We formed this scale by exploratory factor analysis, starting with more than 30 characteristic variables, and tested the resulting three factors with confirmatory factor analysis. The factors and their indicators were then modified to attain convergence and also meaningful factor loadings. We then defined the index as the sum of individual items with equal weight after obtaining a one factor model with meaningful factor loadings in terms of expected sign, statistical significance and width of confidence intervals. At this stage we preferred to give equal weights for the identified variables to create a composite score since we could not find convincing evidence to prioritise one over the other in terms of its potential effect on infant nutritional status in this setting. The scale had a Cronbach alpha value of 0.73 which shows an acceptable level of internal consistency.

### Other potential risk factors

The following characteristics were treated individually within logistic and linear regression analysis while modelling nutritional status of the infants: residence (urban or rural), number of children under the age of 5 years (0, 1, 2+), age of father, age of mother, height of mother, mid-upper arm circumference of mother (MUAC) at recruitment, marital status (polygamous versus non-polygamous), substance use as a binary variable (using khat (*catha edulis Forsk*) at least weekly (it is a widely used stimulant in the Horn of Africa and Arabian Peninsula) or drinking alcohol at least weekly), at least one obstetric complication (prolonged labour (>24 hours), assisted delivery (normal vaginal delivery versus instrumental/Caesarian section), post-partum haemorrhage, post-partum fever), infant gender, immunisation status of infant at two months (yes/no) as an indicator of maternal health-seeking behaviour, history of infant illness in the first two months of life to the extent that the mother thought the baby was going to die (yes/no), birth weight (low (birth weight (BW) < 2500 g), normal (BW ≥ 2500 g), not measured) and early infant feeding practices. Particular focus was given to characteristics that described how the child was fed in the first two months of life: whether the infant was exclusively breast fed in the first two months as reported by the mother at the two month follow-up time-point (yes/no), if the infant was given pre-lacteal food (yes/no), if the infant was given colostrum (yes/no) and the timing of initiation of breast feeding (≤ one hour versus > one hour). We aimed to estimate the crude and adjusted effect of each of these variables upon nutritional status of infants at the ages of six and twelve months.

#### Outcome: Infant nutritional status

Anthropometric measurements taken between five and seven months and between eleven and thirteen months were considered for six and twelve month nutritional indicators, respectively. Since use of standardised scores of weight and length rather than raw measurements makes local and international comparisons easier, growth measurements were standardised to generate z-scores using the 2006 WHO reference population [28]. Height-for-age reflects reduced skeletal growth as the result of repeated undernutrition (or long-standing undernutrition) and weight-for-age reflects both short and long term nutritional deficiencies. Taking the cut-off of -2 z scores, both indices were dichotomised to generate proportion of infants who were stunted (height-for-age z less than -2) and underweight (weight-for-age z less than -2). This resulted in four related nutritional outcome measures, two binary and two continuous. Each of these four outcome variables was considered for regression modelling at six months as well as at twelve months of age.

### Data Management

Data was checked in the field by supervisors and double-entered on the day of collection using Epidata [29]. Women were re-interviewed within one week if data was missing. Ongoing quality checks were performed by the supervisors and the authors (CH and GM).

### Data Analysis

Means and proportions of selected characteristics were used to describe the study participants. In evaluating potential risk factors of infant nutritional status at both time points, logistic regression for binary outcomes and linear regression for continuous outcomes were used. Unadjusted and adjusted odds ratios from logistic regression and unstandardised regression coefficients from linear regression with corresponding 95% confidence intervals were used to assess the significance and the magnitude of the effect of a given exposure. In building the fully adjusted model, the following two steps were followed: (1) estimate the fully adjusted model for all variables presented in respective tables except for the four variables listed under "feeding practices", (2) to obtain the independent effect of each feeding practice, we re-estimated the model in (1) including also the feeding practice variable of interest. This generated (a) the effect of each variable adjusted for all others except feeding practice variables, and (b) the effect of each feeding practice variable adjusted for all other variables but not for the remaining feeding practice variables. We did not adjust each infant feeding practice for the others because of collinearity and to enable us to obtain the independent effect of each feeding practice upon undernutrition. To maximise the use of available data, birth weight was included in all models as a three category variable (normal (BW > = 2500 gm), low birth weight (BW <2500 gm) and birth weight not recorded). All data analysis was carried out using STATA version 10 [30].

## Results

### Cohort characteristics

**Figure 1:**
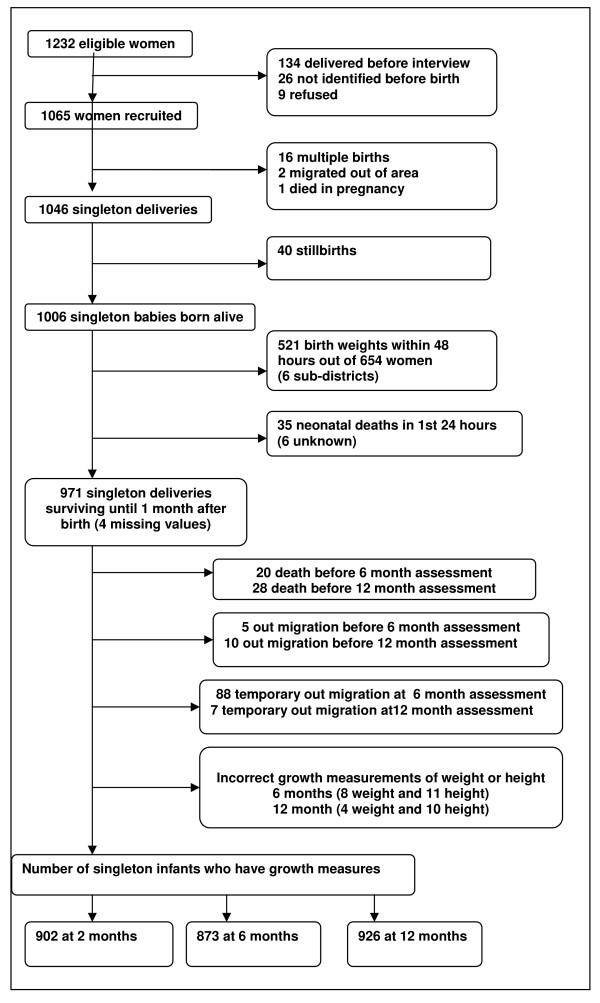
**Follow-up of the P-MaMiE cohort from screening up to one year postnatal**.

Initial recruitment and detailed description of follow-up is presented in figure 1. From the cohort of 1065 women we now present data on 873 infants (82.0%) followed up to six months of age and 926 infants (86.9%) followed up to the 12 months of age. The main causes of attrition were neonatal mortality (n = 35), stillbirth (n = 40), and multiple births (15 twins and 1 triple birth). Temporary (n = 95) and permanent (n = 10) out-migration also contributed to loss to follow-up. Nineteen cases at six months and 14 cases at twelve months were excluded due to measurement error on the outcome and flagged by the Anthro software [28] as being outliers during standardisation of growth measurements.

Ninety nine percent of the women were married and all younger (mean age 27 years, sd = 6.2 years) than their husbands (mean age 36 years, sd = 8.8 years) (Table 1). The majority of women were non-literate (85%), reported their occupation as housewives or farming (87%), and belonged to the Meskan ethnic group (46%). Comparatively, the non-literacy rate was lower among the husbands (32%). Most of the women reported having protected water (69%) and a toilet facility (63%) but few of them disposed of rubbish in a sanitary way (22%).

**Table 1:** Selected background characteristics of P-MaMiE study participants

Characteristics	At 6 month follow-up	At 12 month follow-up
	N (%)/Mean (SD)	N (%)/Mean (SD)
***Maternal Characteristics ***		
Religion:		
Muslim	674 (77.2)	722 (78.0)
Orthodox Christian	133 (15.3)	139 (15.0)
Protestant	56 (6.4)	55 (5.9)
Catholic	10 (1.2)	10 (1.1)
Ethnicity:		
Meskan	404 (46.3)	436 (47.1)
Mareko	119 (13.7)	123 (13.3)
Silti	200 (22.8)	218 (23.5)
Sodo	69 (7.9)	68 (7.3)
Others	81 (9.3)	81 (8.8)
Currently married	865 (99.1)	917 (99.0)
Occupation		
Housewife or farming	759 (87.1)	805 (87.1)
Self or paid employee	112 (12.9)	119 (12.9)
Age (years)	26.8(6.2)	26.9(6.2)
Educational status		
Literate	134 (15.4)	141 (15.2)
Non-literate	739 (84.7)	785 (84.8)
***Household characteristics***		
Age of husband (years)	36.0(8.8)	36.1(8.9)
Educational status of husband		
Literate	594 (68.4)	632 (68.7)
Non-literate	274(31.6)	288 (31.3)
Main source of water		
Protected supply	601 (69.0)	644 (69.7)
Unprotected supply	270 (31.0)	280 (30.3)
Sanitary condition		
Have toilet facilities	553 (63.3)	582 (62.9)
No proper toilet facilities	320 (36.7)	344 (37.2)
Rubbish disposal		
Buries, burns or others	192 (22.0)	200 (21.6)
Disposes on field	680 (78.0)	725 (78.4)
***Traditional surgical practices before two months of age***		
Uvulectomy:		
Performed	16 (1.8)	16 (1.7)
Not Performed	853 (98.2)	905 (98.3)
Circumcision of girls:		
Performed	6 (1.4)	7 (1.5)
Not Performed	415 (98.6)	447 (98.5)
Circumcision of boys:		
Performed	187 (41.6)	196 (41.9)
Not Performed	262 (58.4)	272 (58.1)

The prevalence of regular khat chewing during pregnancy (28%) was significantly higher than during the postnatal period (21%) (OR = 1.84; 95% CI: 1.38, 2.47). The prevalence of regular alcohol consumption was generally low among the study participants and reduced significantly during the postnatal period compared to pregnancy (1.6% & 5.1%; p < 0.001). In this cohort, harmful traditional practices were relatively rare with a low prevalence of performing uvulectomy (1.8% before two months of age and 4.8% before one year of age), female circumcision (1.5%) before two months of age and milk teeth extraction (3.9%) in the first year of infancy.

Fifty four percent of women attended antenatal care, 90% delivered at home and only 24% of deliveries were attended by trained personnel at any level. Despite this, a high proportion of mothers reported giving colostrum to their newborn (82%), 32% initiated breastfeeding within the first hour and 16% reported giving pre-lacteal food to their new born. At the age of two months 59% of the infants had already received at least one type of vaccination: 53% Polio; 29% DPT, and 35% BCG.

At one year of age 99.6% of the infants were breastfeeding. At the six month anthropometric assessment 33.4% of the mothers reported that their infants were currently ill; this prevalence was reduced to 30.6% during twelve month growth assessment. During the same follow-up 95.2% of the mothers reported at least one infant illness episode since birth, 60.0% of whom had thought that their baby was going to die because of the severity of the episode. Introduction of supplementary feeding occurred for 33.8 of infants by six months, 36.8% during 7-9 months and 28.3% after 9 months.

### Prevalence of undernutrition and risk factors

**Figure 2:**
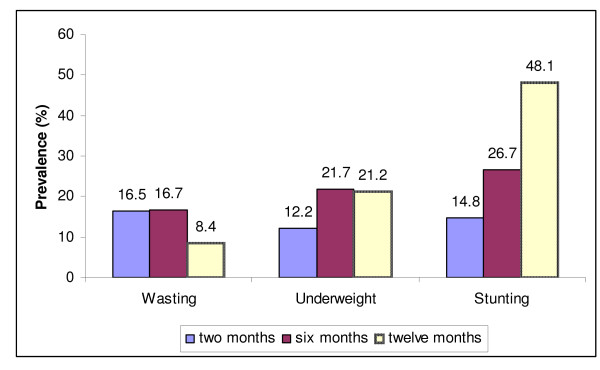
**Prevalence of undernutrition of infants at two, six and twelve months of age in the P-MaMiE study**.

The prevalence of infant undernutrition increased throughout the first year of life (Figure 2). The overall prevalence of stunting and underweight were 14.6% and 10.8% at the age of two months, 26.7% and 21.7% at the age of six months and 48.1% and 21.2% at the age of twelve months, respectively. The prevalence of undernutrition was significantly higher amongst infants who were male, born with low birth weight, residing in rural areas, born from under-nourished mothers, had older parents, had non-literate parents, were living in a less hygienic environment, scored higher on the poverty index, and for whom initiation of breastfeeding was delayed or colostrum was not given. (Table 2 and Table 3)

**Table 2:** Nutritional status of infants at six and twelve months of age stratified by selected maternal, infant and environmental characteristics

***Selected *background characteristics**	**6 month**				**12 month**			
	**Underweight**		**Stunted**		**Underweight**		**Stunted**	
	
	**Number (%)**	**p-value**	**Number (%)**	**p-value**	**Number (%)**	**p-value**	**Number (%)**	**p-value**
	
**Sex**								
Male	119(26.9)	0.000	150(33.9)	0.000	107(23.0)	0.173	266(57.0)	0.000
Female	69(16.4)		80(19.1)		88(19.3)		175(39.0)	
**Birth weight**								
Normal	108(20.4)	0.000	121(23.0)	0.000	108(20.4)	0.000	121(23.0)	0.008
Low	23(51.1)		14(31.8)		23(51.1)		14(31.8)	
Missing	57(19.7)		95(32.7)		57(19.7)		95(32.7)	
**Residence**								
Rural	173(23.4)	0.005	196(26.5)	0.795	181(22.7)	0.002	398(50.2)	0.002
Urban	15(12.1)		32(25.6)		14(11.3)		43(35.0)	
**Under five children**								
None	44(25.1)	0.429	48(27.1)	0.873	28(14.8)	0.057	84(44.9)	0.590
Only one	91(20.4)		121(27.2)		107(22.8)		230(49.4)	
Two or more	58(21.8)		61(25.4)		60(22.8)		127(48.3)	
**Sanitation facility**								
Toilet available	94(17.2)	0.000	133(24.4)	0.047	112(19.4)	0.088	257(44.6)	0.005
Open field	94(29.7)		97(30.6)		83(24.1)		184(54.1)	
**Rubbish disposal**								
Burn	29(15.1)	0.011	53(27.6)	0.752	34(17.1)	0.111	76(38.2)	0.001
Open field	159(23.7)		177(26.5)		161(22.3)		365(51.0)	
**Water source**								
Protected	117(19.6)	0.020	175(29.5)	0.006	104(16.2)	0.000	285(44.7)	0.001
Unprotected	71(26.7)		55(20.6)		91(32.6)		156(56.5)	
**Initiation of breast feeding**								
< = 1 hour	47(17.1)	0.023	69(25.2)	0.439	55(19.0)	0.281	142(49.5)	0.655
>1 hour	138(24.0)		159(27.7)		137(22.2)		294(47.9)	
**Colostrums**								
Given	142(20.5)	0.040	189(27.4)	0.505	153(20.6)	0.318	363(49.2)	0.313
Denied	45(28.0)		40(24.8)		40(24.1)		74(44.9)	
**Pre-lacteal feeding**								
Given	9(22.5)	0.934	10(25.0)	0.766	8(19.1)	0.728	21(48.8)	0.946
Not given	178(22.0)		219(27.1)		184(21.3)		414(48.3)	
**Breastfeeding at two months**								
Exclusive	158(21.9)	0.835	192(26.8)	0.916	167(21.6)	0.394	361(47.1)	0.137
Non-exclusive	30(21.1)		38(26.4)		27(18.5)		78(53.8)	
**Maternal education**								
Literate	38(22.0)	0.007	52(30.2)	0.239	36(19.5)	0.529	79(43.7)	0.176
Non-literate	150(21.7)		178(25.8)		159(21.6)		362(49.3)	
**Father's education**								
Literate	117(19.8)	0.044	151(25.8)	0.378	128(20.3)	0.407	292(46.7)	0.183
Non-literate	70(25.9)		78(28.7)		65(22.7)		146(51.4)	

**Table 3:** Mean (standard deviation) of selected background parental characteristics stratified by infants' age and nutritional status

Background characteristics	Underweight			Stunted		
	Yes	No	p-value	Yes	No	p-value
***Six month time point***						

Maternal age	27.2(6.3)	26.7(6.2)	0.336	26.8(6.2)	26.9(6.2)	0.896
Age of father	37.1(9.2)	35.8(8.7)	0.071	36.0(8.8)	36.0(8.9)	0.994
Maternal MUAC	24.3(1.9)	24.8(2.2)	0.002	24.5(2.0)	24.8(2.3)	0.131
Poverty index	7.6(1.8)	7.2(2.0)	0.013	7.1(2.1)	7.3(2.0)	0.261
Sanitary index	1.7(1.0)	1.4(1.0)	0.000	1.4(1.0)	1.5(1.0)	0.665
Maternal support	1.10(0.97)	1.09(1.09)	0.866	1.09(1.05)	1.10(1.07)	0.967
Maternal autonomy	1.17(1.86)	1.14(1.87)	0.785	1.32(1.98)	1.08(1.83)	0.094

***Twelve month time point***						

Maternal age	28.0(6.4)	26.6(6.2)	0.004	27.2(6.1)	26.6(6.4)	0.165
Age of father	37.4(9.7)	35.7(8.6)	0.016	36.4(8.8)	35.8(8.9)	0.333
Maternal MUAC	24.4(2.0)	24.8(2.2)	0.013	24.6(2.1)	24.8(2.2)	0.105
Poverty index	7.5(1.7)	7.2(2.0)	0.078	7.4(1.8)	7.1(2.1)	0.022
Sanitary index	1.7(1.0)	1.4(0.9)	0.000	1.6(0.9)	1.3(0.9)	0.000
Maternal support	1.17(1.03)	1.07(1.08)	0.254	1.13(1.06)	1.05(1.07)	0.242
Maternal autonomy	1.18(1.88)	1.11(1.83)	0.650	1.06(1.82)	1.15(1.83)	0.444

MUAC = Maternal mid-upper circumference

### Comparison of the cohort with 2006 WHO child growth standards

Compared to the 2006 WHO child growth standards, which have a mean of zero and standard deviation of one, infants in this cohort in the first year of life were significantly lighter as reflected in their mean (95% CI) weight-for-age z score at two months (-0.54; -0.62 to -0.47), six months (-1.09; -1.17 to -1.00), twelve months (-1.06; -1.14 to -0.98) and shorter as reflected in their mean (95% CI) height-for-age z score at two months (-0.34; -0.45 to -0.23), six months (-1.08; -1.19 to -0.98) and twelve months of age (-2.04; -2.14 to -1.94).

### Bivariate analysis

Results from bivariate analysis of each covariate and the growth outcomes are presented in Tables 4 and 5. Living rurally, having an older parent, scoring more on the sanitary scale and poverty index, being of male gender, low birth weight, not receiving colostrum, delayed initiation of breastfeeding, and lower maternal mid-upper arm circumference (MUAC) were significant predictors of underweight at the age of six months although the significance of MUAC was marginal. Only male gender and being vaccinated before two months of age were associated with stunting. At the age of twelve months, male gender, not being given colostrum, and delayed initiation of breastfeeding were no longer risk factors for being underweight, but having other children under the age of five became independently associated, along with older parental age, rural residence, poor sanitary conditions, low birth weight and maternal undernutrition. With respect to stunting at twelve months of age the risk factors were rural residence, increased values of poverty index and poor sanitary scores, male gender and low birth weight. There was very little difference in the magnitude and confidence intervals of the effect size of parental age on underweight and stunting at six and at twelve months of age

**Table 4:** Predictors of infant undernutrition at 6 months of age in Butajira Birth cohort, Ethiopia (n = 873)*

	Underweight			Stunting		
	N (%) or Mean(SD)	Crude OR (95% CI)	Adjusted OR (95% CI)	N (%) or Mean(SD)	Crude OR (95% CI)	Adjusted OR (95% CI)
***Feeding practices individually adjusted for full model***						
Non-exclusive breast-feeding at 2 months	142 (16.5)	0.95(0.61,1.48)	0.94(0.57,1.53)	144(16.8)	0.98(0.65,1.47)	0.80(0.51,1.24)
No pre-lacteal food	810 (95.3)	0.97(0.45,2.07)	1.08(0.44,2.63)	806(95.3)	1.12(0.54,2.32)	1.21(0.54,2.72)
Colostrums not given	160 (18.8)	1.50(1.02,2.22)	1.06(0.67,1.66)	160(18.9)	0.87(0.59,1.30)	0.85(0.55,1.31)
Breast feeding delayed for 1 hour	576 (67.8)	1.53(1.06,2.21)	1.40(0.93,2.12)	574(67.8)	1.14(0.82,1.58)	1.13(0.79,1.63)

**Fully adjusted model**						

***Characteristics of mother***						
Age (years)	26.8(6.2)	1.01(0.99,1.04)	1.01(0.97,1.05)	26.9(6.2)	1.00(0.97,1.02)	0.99(0.96,1.03)
Height (metres)	1.58(0.06)	0.99(0.97,1.02)	1.00(0.97,1.03)	1.58(0.06)	1.01(0.98,1.03)	1.00(0.98,1.03)
Mid upper arm circumference (cm)	24.7(2.1)	0.88(0.81,0.96)	0.87(0.80,0.96)	24.7(2.1)	0.95(0.88,1.02)	0.94(0.87,1.02)
Being in polygamous marriage	153 (17.9)	1.01(0.67,1.54)	0.89(0.52,1.54)	153(17.7)	0.98(0.66,1.45)	1.06(0.64,1.76)
Autonomy scale (0-5)	1.1(1.9)	1.01(0.93,1.10)	1.06(0.96,1.17)	1.1(1.9)	1.07(0.99,1.16)	1.11(1.02,1.21)
Use khat and/or alcohol	101(11.7)	0.76(0.44,1.29)	0.84(0.47,1.52)	100(11.6)	0.90(0.56,1.46)	1.15(0.69,1.93)
Had at least one obstetric complication	534(63.9)	0.82(0.59,1.15)	0.97(0.66,1.42)	532(63.9)	0.82(0.60,1.12)	0.86(0.61,1.21)
***Household characteristics ***						
Urban residence	124 (14.4)	0.45(0.26, 0.80)	0.54(0.23,1.27)	123(14.3)	1.06(0.69,1.62)	0.54(0.27,1.09)
Number of under 5 children:						
0	174 (20.1)	1	1	176(20.4)	1	1
1	447 (51.7)	0.76(0.50, 1.15)	0.54(0.33,0.89)	445(51.7)	1.00(0.68,1.48)	0.95(0.60,1.51)
≥ 2	243 (28.1)	0.83(0.53, 1.31)	0.73(0.43,1.27)	240(27.9)	0.92(0.59,1.42)	0.99(0.59,1.66)
Age of father in years	36.1(8.8)	1.02(1.00,1.04)	1.03(1.00,1.06)	36.0(8.9)	1.00(0.98,1.02)	1.01(0.98,1.04)
Poverty index (0 - 11)	7.3(2.0)	1.02(1.02,1.22)	0.97(0.85,1.11)	7.3(2.0)	0.96(0.89,1.03)	0.94(0.83,1.06)
Poor sanitary condition (0 -3)	1.5(1.0)	1.46(1.23,1.74)	1.35(1.09,1.68)	1.5(1.0)	0.97(0.83,1.13)	0.96(0.79,1.16)
Level of social support (0 - 4)	1.1(1.1)	1.01(0.87,1.18)	1.00(0.84,1.18)	1.1(1.1)	1.00(0.86,1.15)	1.00(0.86,1.16)
***Characteristics of index child***						
Female gender	422 (48.8)	0.53(0.38,0.74)	0.49(0.34,0.71)	420(48.8)	0.46(0.33,0.63)	0.40(0.28,0.56)
Not immunised at two months	352 (40.8)	0.95(0.68,1.32)	0.93(0.64,1.34)	351(40.9)	0.72(0.53,0.99)	0.71(0.51,1.00)
Severe illness in the first 2 months	184 (21.4)	1.12(0.76,1.65)	0.90(0.58,1.40)	184(21.4)	1.18(0.82,1.69)	1.03(0.69,1.53)
Birth weight:						
Normal (> = 2500 g)	530 (61.3)	1	1	527(61.2)	1	1
Low (<2500 g)	45 (5.2)	4.09(2.19,7.61)	4.12(2.06,8.21)	44(5.1)	1.57(0.80,3.05)	1.69(0.81,3.50)
Not measured	289 (33.5)	0.96(0.67,1.37)	0.98(0.66,1.46)	290(33.7)	1.63(1.18,2.24)	1.50(1.06,2.13)

* this number slightly vary in some variables due to missing values

**Table 5:** Predictors of infant undernutrition at 12 months of age in Butajira Birth cohort, Ethiopia (n = 926)*

Characteristics considered	Underweight			Stunting		
	n (%) or Mean(SD)	Crude OR (95% CI)	Adjusted OR (95% CI)	N (%) or Mean(SD)	Crude OR (95% CI)	Adjusted OR (95% CI)
***Feeding practices individually adjusted for full model***						
Non-exclusive breast-feeding at 2 months	146(15.9)	0.82(0.52,1.29)	0.87(0.53,1.42)	145(15.9)	1.31(0.92,1.87)	1.46(0.97,2.20)
No pre-lacteal food	864(95.4)	1.15(0.52,2.53)	1.02(0.42,2.48)	857(95.2)	0.98(0.53,1.81)	0.99(0.50,1.95)
Colostrums not given	166(18.3)	1.22(0.82,1.82)	1.04(0.66,1.63)	165(18.3)	0.84(0.60,1.18)	0.78(0.53,1.15)
Breast feeding delayed for 1 hour	618(68.1)	1.21(0.85,1.72)	1.20(0.81,1.78)	614(68.2)	0.94(0.71,1.24)	0.85(0.62,1.17)

**Fully adjusted model**						

***Characteristics of mother***						
Age (years)	26.9(6.2)	1.04(1.01,1.06)	1.04(1.00,1.08)	26.9(6.2)	1.01(0.99,1.04)	1.02(0.99,1.06)
Height (metres)	1.58(0.06)	1.00(0.97,1.02)	1.00(0.97,1.03)	1.58(0.06)	0.99(0.97,1.01)	0.99(0.97,1.02)
Mid upper arm circumference (cm)	24.7(2.1)	0.91(0.84,0.98)	0.89(0.81,0.97)	24.7(2.1)	0.95(0.89,1.01)	0.95(0.89,1.02)
Being in polygamous marriage	162(17.6)	0.90(0.59,1.38)	0.77(0.45,1.33)	162(17.7)	1.06(0.76,1.49)	1.19(0.76,1.87)
Autonomy scale (0-5)	1.1(1.8)	1.02(0.94,1.11)	1.04(0.95,1.15)	1.1(1.8)	0.97(0.91,1.04)	1.00(0.92,1.08)
Use khat and/or alcohol	104(11.3)	0.70(0.41,1.22)	0.81(0.45,1.47)	104(11.4)	0.77(0.51,1.16)	0.81(0.51,1.28)
Had at least one obstetric complication	573(64.5)	0.91(0.65,1.27)	1.03(0.71,1.49)	571(64.7)	0.86(0.65,1.14)	0.92(0.68,1.26)
***Household characteristics ***						
Urban residence	124(13.5)	0.43(0.24,0.78)	0.38(0.16,0.90)	123(13.4)	0.53(0.36,0.79)	0.52(0.28,0.99)
Number of under 5 children:						
0	189(20.5)	1	1	187(20.4)	1	1
1	470(51.0)	1.69(1.07,2.67)	1.33(0.77,2.30)	466(50.9)	1.20(0.85,1.68)	0.96(0.63,1.46)
≥ 2	263(28.5)	1.70(1.04,2.79)	1.75(0.98,3.14)	263(28.7)	1.15(0.79,1.67)	1.19(0.76,1.87)
Age of father in years	36.0(8.8)	1.02(1.00,1.04)	1.02(0.98,1.05)	36.1(8.9)	1.01(0.99,1.02)	1.00(0.97,1.02)
Poverty index (0 to 11)	7.3(2.0)	1.08(0.99,1.17)	0.85(0.74,0.97)	7.3(2.0)	1.08(1.01,1.15)	0.98(0.88,1.09)
Poor sanitary condition (0 to 3)	1.5(1.0)	1.45(1.22,1.72)	1.39(1.13,1.73)	1.5(0.9)	1.37(1.19,1.58)	1.30(1.08,1.55)
Higher level of social support (0 to 4)	1.1(1.1)	1.09(0.94,1.26)	1.07(0.91,1.26	1.1(1.1)	1.08(0.95,1.22)	1.06(0.93,1.22)
***Characteristics of index child***						
Female gender	456(49.5)	0.80(0.58,1.10)	0.86(0.60,1.23)	449(49.0)	0.48(0.37,0.63)	0.46(0.34,0.62)
Not immunised at two months	381(41.5)	1.22(0.89,1.69)	1.15(0.81,1.65)	379(41.6)	0.96(0.74,1.25)	0.97(0.72,1.30)
Severe illness in the first 2 months	193(21.0)	1.13(0.77,1.66)	1.17(0.76,1.79)	192(21.1)	0.91(0.66,1.26)	0.83(0.58,1.19)
Birth weight:						
Normal (> = 2500 gm)	570(61.8)	1	1	568(62.0)	1	1
Low (<2500 gm)	43(4.7)	3.08(1.62,5.85)	3.00(1.47,6.13)	41(4.5)	2.71(1.39,5.28)	2.88(1.38,6.03)
Not measured	309(33.5)	1.23(0.88,1.73)	1.28(0.87,1.87)	307(35.5)	1.94(1.46,2.57)	2.18(1.59,3.01)

* this number slightly vary in some variables due to missing value

### Multivariable analysis

The adjusted effects of covariates on the infant growth outcomes are presented in Tables 4 and 5. Poor sanitation and low birth weight were associated with being underweight at both time points and with stunting at twelve months of age. Male gender was associated with stunting at both time-points and being underweight at six months. Rural residence was associated with stunting and underweight at twelve months of age but not associated with infant undernutrition at six months of age. Infants with older parents were more likely to be disadvantaged at both time points although the effect size associated with one year increase in parental age was relatively small. Maternal nutritional status was associated with being underweight at both time-points but not with stunting. Having a sibling aged under five and a higher score on the poverty scale were protective against becoming underweight at the six and twelve month time points, respectively, with marginal statistical significance of the latter. A higher value on the autonomy scale was associated with stunting at six month although the statistical significance of the effect was marginal.

Linear regression using weight-for-age z score and height-for-age z score as continuous indices at both the six month and twelve month time-points did not yield any major differences from use of the dichotomised anthropometric indices (results available from the first author on request).

## Discussion

In this study we have reported on the prevalence and predictors of infant undernutrition in a predominantly rural, population-based prospective birth cohort. Such studies are rare in resource poor countries given the lack of vital registration. Sub-optimal early feeding practices were not associated with nutritional status of infants except in unadjusted analysis where witholding colostrum and delaying initiation of breastfeeding for more than an hour were risk factors for underweight at six months of age. There was a clear and striking picture of infant undernutrition from as early as two months of age that worsened continuously to the age of one year. Girls fared better than boys implying vulnerability of the latter to the existing harsh environment. We were also able to replicate findings of important risk factors from previous studies on infant undernutrition. We found rural residence, low birth weight, poor sanitary conditions, maternal undernutrition, male gender, increased parental age and having two or more children other than index child aged under five to be the main risk factors for infant undernutrition in this study area. Scales of maternal autonomy, availability of support the mother and poverty do not seem to have a clear association with infant undernutrition in this setting, probably due to lack of variability in the study area with respect to these factors. Having older parents seems to be a risk factor for undernutrition which could possibly be explained by large family size with limited resources to be distributed among the large number of siblings.

The strength of this study comes from its design and use of both binary and continuous variables to model infant nutritional status. Unlike most previous studies which were cross-sectional, we were able to measure exposure variables prospectively, free of information bias, and to assess the stability of the association across the neonatal and infant periods of development. We were also able to compare the patterns of association with the outcome of interest when analysed as binary variable (i.e. underweight and stunting) for easy practical interpretation and as a continuous variable (i.e. weight-for-age z score and height-for-age z score) to maximize statistical power. Unlike cross-sectional studies that rely on the recall of parents to determine infant age, which would be difficult in our study setting, we have calculated age based on the contemporaneously recorded date of birth. This information is not routinely available given the low percentage of women in Ethiopia who give birth in clinics or hospitals.

A number of limitations can be identified that might have affected our findings. Most of the risk factors were obtained from maternal report as there was no other means of obtaining that information, and as such could have been subject to recall bias. However, the outcome was measured prospectively and so maternal recall bias is less likely to have affected the observed associations. Several research workers were involved in measuring height and weight outcomes, which might have introduced measurement errors. Inter-rater reliability was not assessed formally. The training, close follow-up by first authors (GM and CH), periodic quality-control checks and re-measuring of any suspected error cases within one week, should have minimised measurement errors. Any resulting error is likely to be random, and would have had the effect of reducing the size of any genuine effect towards the null. Only measuring birth weight in selected sub-districts might have influenced some of the associations, although non-measured birth weights were missing at random [25]. To account for this we include a three category variable (normal, low birth weight, not measured) in our analysis and the effect of low birth weight was in the expected direction, increasing our confidence in our findings. Although the two month window used to define the outcome could potentially compromise the precision of prevalence estimates of undrnutrition at six and twelve month of age, comparison of the prevalence of underweight and stunting for infants measured within and outside the target one month window did not reveal significant difference. Mortality accounted for the majority of infants lost to follow-up; a higher prevalence of undernutrition within this group might have biased our point estimates. Another limitation of the study is that participating women were not able to correctly report their LMP

The prevalence of undernutrition increases progressively in Ethiopia from early infancy, peaking in the late months of the second year of age and thereafter starting to stabilise. The prevalence of stunting starts to exceed that of underweight when undernutrition starts to stabilise [15]. The current finding follows the same increasing trend but the prevalence of stunting overtook that of underweight as early as two months of age. This difference is not unexpected in light of the varying degree of undernutrition across different geographical regions of the country [13,15]. However, for 48% of infants to be stunted at one year of age while 99.6% of them are still breastfeeding is of concern. The national prevalence of stunting is 32.7% and 46.3% among 9-11 months old and 12-17 months old, respectively [15]. In Burkina Faso [31] the prevalence of stunting at the mean age of 5 months is less than 2.0% and in a semi-urban population in Uganda the prevalence of stunting among 0-11 month old infants is 16.7% [20]. A prevalence of stunting comparable to the current study was reported among 0-12 months old in India [19]. One possible explanation for the high prevalence of stunting at one year could be due to the late introduction of supplementary food with low nutritional quality [13]. Similarities and differences across different studies could be attributed to the underlying socioeconomic and socio-cultural conditions. The Meskan, Mareko and Silti districts have been subject to drought in recent years. During the study period, bi-annual screening for severely undernourished infants and pregnant/lactating women was being conducted in order to provide food supplements. The high prevalence of stunting is therefore not unexpected.

Like other African countries [32] breastfeeding at one year of age is a norm in Ethiopia [15] and 99.6% of the current study participants were breastfeeding at one year follow-up. However, adherence to the optimal breastfeeding practices [33] that could reduce infant morbidity and enhance growth [34,35] is still low in Ethiopia [36]. Several investigators [20,19] have reported an increased risk of infant undernutrition resulting from sub-optimal feeding during early infancy (discarding of colostrum, delayed initiation of breast feeding, pre-lacteal feeding and non-exclusive breastfeeding). However, as in our study, other investigators did not replicate these findings [37,31]. In Bangladesh, food secure households were more likely to practice sub-optimal infant feeding at the age of 3-6 months [38] but the prevalence of undernutrition was significantly lower in this group compared to food insecure households [39]. In Egypt pre-lacteal feeding was associated with an increased risk of diarrhoea and early introduction of supplementary food [40]. In the current study, the proportion of women delaying initiation of breastfeeding for more than one hour is comparable to the national figure but the proportion of non-excusive breastfeeding at the age of two months and pre-lacteal feeding practices are relatively low [15,36]. The proportion of infants who were given colostrum in the current study is higher than studies from Burkina Faso [31] and India [19]. One possible explanation for the current negative finding might be a lack of power to detect an effect due to the low prevalence of sub-optimal feeding practices. An absence of any significant effect of these practices in the study area could also be a possibility although the observed significant effect of withholding colostrum and delaying initiation of breastfeeding in the unadjusted model makes this explanation less likely.

Maternal MUAC as a measure of nutritional status in pregnancy had a significant protective effect upon an infant being underweight at both six and twelve month assessments, even after adjusting for possible risk factors, but was not associated with stunting at either time-point. In a cross-sectional sample of infants aged 5-11 months in two rural villages of Ethiopia, maternal height, triceps skin fold thickness and zinc concentration in beast milk were associated with stunting but current weight and MUAC were not associated with stunting [41]. Maternal height and weight gain during pregnancy have previously been found to be significant predictors of severe stunting of infants at one year of age [42]. In the current study we do not have data to investigate either the effect of weight gain during pregnancy or the effect of the content of breast milk. Stunting is thought to result from both the nutritional experience of the individual over a period of time, as well as the nutritional status of the parents, particularly the mother, with the maternal effect mainly acting through birth weight [9] and possibly birth length [43]. One possible explanation for the observed non-significant effect of maternal nutritional status on stunting could be the result of strong negative effects of competing postnatal environmental risk factors which might have diluted any meaningful effect. The inverse relationship between maternal MUAC and infant underweight suggests that interventions that improve the nutritional status of pregnant women in this setting may also improve infant weight gain during infancy, over and above any effect mediated through birth weight

In this study, the mean poverty index score was significantly higher for mothers whose infants were undernourished compared to mothers whose infants were not. However, the mean maternal autonomy score and mean score of availability of support to the mother did not differ between the two groups of women. In the adjusted model none of the three composite scores was significantly related to infant undernutrition. As the primary caregiver, it is probable that maternal autonomy is critical for the overall wellbeing of infants [44] although it did not show significant protective effect against infant undernutrition in this study. In a cohort study in Pakistan [45] maternal financial autonomy was not significantly associated with nutritional status of infants after adjusting for other risk factors but higher socioeconomic status was associated with better nutritional status. In India, better nutritional status was associated with increased maternal autonomy to use household finances and increased freedom to go to the market [21,46]. In another urban sample of under five children in India, of which 54.4% were 0-12 months old, the prevalence of underweight was higher among middle income groups relative to the lower income group [19]. Socioeconomic differentials [47,48] and the amount of social support during pregnancy [49,50] influence foetal growth, with an unfavourable postnatal environment aggravating the situation during infancy and beyond [51]. A significant reduction in undernutrition among under five children in Brazil was attributed to the overall economic development in the country [52]. After adjusting for the effect mediated through birth weight these constructs might have only exerted a small influence in our study area and we may not have been power to detect such small effects. In other words, the overall level of poverty in the current study area, with very low variability of socio-economic status amongst participants and a high rate of non-literacy rate that might not have allowed mothers to exercise their reportedly high autonomy for better childcare practices, might explain the current non-significant findings beyond that mediated through birth weight.

Low birth weight was a significant predictor of stunting and underweight at twelve months and underweight at six months of age. This is in line with a previous study from Pakistan reporting a higher risk of stunting at one year of age for low birth weight infants [53]. In the absence of birth weight and length, neonatal weight and length were the most important predictors of child nutritional status in Indonesia [43]. The effect of low birth weight we have observed at six months might be due to their disadvantage in utero [51,54] or might have been mediated through recurrent infant illness episodes [55-57,51].

Living in poor sanitary conditions significantly increased the risk of an infant being underweight and stunted at 12 months of age and significantly predicted underweight at six months of age. Although these findings might be expected, improving sanitation is an important area for increased intervention to reduce undernutrition [4]. Other studies have not always found such an association [58,59]. Poor sanitation most probably exerts its effect on infant nutritional status by increasing the risk of infectious illnesses [43]. Therefore variation in factors affecting the prevalence of infectious disease between settings may explain the observed difference. Ethiopian mothers have been shown to understand the role of poor sanitation in child undernutrition but tend to attribute child undernutrition to their poor economic status and not something amenable to changes in their sanitation behaviours [58].

In the present study boys were more likely to be underweight at six months and more likely to be stunted both at six and at twelve months. A significantly higher prevalence of undernutrition amongst boys as compared to girls is consistent with other Ethiopian studies [14,15,58] and other African studies [60,42,59-64], and more pronounced in the lowest socioeconomic groups [63]. These results have not, however, been confirmed in a number of other studies [65-67,41]. The higher prevalence of anaemia among boys and in infants from poor households in a nationally representative Ethiopia study [15] is in keeping with the current finding. The existing vulnerability of boy babies that is seen in all cultures, and may partly have a genetic basis [68], may explain the observation. Gender preference and differential feeding practices or neglect of boys are unlikely to be the reason, with published studies in Ethiopia tending to show that it is female infants who usually receive less food than their male counterparts [69,70]

Rural residence significantly predicted underweight and stunting at twelve months of age in line with other African countries [71]. This was explained mainly by urban-rural socioeconomic differences. A recent demographic and health survey has revealed systematic inequality in various measures between urban and rural settings including undernutrition [15] confirming the result of four repeated countrywide surveys in Ethiopia which have also reported a higher prevalence of undernutrition among rural populations [14]. Discrepancy in the source of income, exposure to the knowledge of appropriate weaning food, and purchasing power for proper weaning food, all favouring the urban setting, could explain some of the observed differences in the current study. Overall Ethiopian mothers seem to associate undernutrition of their infants with shortage of food [58]. The higher prevalence of maternal undernutrition in rural areas [15] is another possible explanation for the current finding although the finding is independent of maternal mid-upper arm circumference and maternal height. Differential micronutrient intake in the two settings is less likely to be an explanation, although consumption of low levels of micronutrient rich food in general might explain part of the high prevalence of undernutrition in the whole birth cohort [15].

Having one previous child aged less than 5 years was found to be protective of being underweight at six months of age which might be explained by the child caring experience of the mother with her first child. There was a statistically non-significant but consistent association with underweight and stunting at six and twelve months of age showing an increased risk of undernutrition with an increased number of under five children prior to index child. A regional survey within Ethiopia [23] and a study in Vietnam [72] have found a direct relationship between number of under five children and undernutrition, which may be explained by the negative effect of short birth intervals (i.e. explained by more number of under five children), on quality of maternal child caring practices. Limited financial resources of the household versus an increase of demand for that resource, including good parenting practices [33], might also explain the association

## Conclusions

In our study infant undernutrition was common and progressively worsened throughout the first year of life. After adjusting for other risk factors, none of the measured early infant feeding practices were associated with undernutrition, suggesting that interventions directed only at these maternal behaviours may have a limited impact in this setting. However, interventions targeted at improving maternal nutritional status during pregnancy might reduce infant undernutrition by increasing birth weight and improving the quantity and quality of breast milk. It might be possible to minimise the rural-urban difference in infant undernutrition by empowering mothers living in rural areas with skills of optimal parenting practices and increasing accessibility of health services. Investment in improving basic sanitary conditions and devising mechanism to improve maternal under-nutrition should be the focus for short-term strategies to reduce infant undernutrition in this setting.

## Competing interests

The authors declare that they have no competing interests.

## Authors' contributions

CH, MP, AA conceived the idea; CH designed the study; GM and CH coordinated data collection and data entry; GM analysed the data and drafted the manuscript, CH, MD, FT, BW, MP, MT, critically commented on the draft manuscript, all authors contributed to the interpretation of results and approved the final manuscript

## Pre-publication history

The pre-publication history for this paper can be accessed here:

http://www.biomedcentral.com/1471-2458/10/27/prepub
